# *trans*-Di­aqua­tetra­kis­(tetra­hydro­furan-κ*O*)iron(II) μ-carbonyl-tetra­deca­carbonyl­tetra­chlorido-μ-di­methyl­silanediolato-tetra­galliumtetra­iron(7 *Ga*–*Fe*)(*Fe*–*Fe*) tetra­hydro­furan tetrasolvate

**DOI:** 10.1107/S2414314624006205

**Published:** 2024-06-28

**Authors:** Mary Elizabeth Demmin, Cary Bauer, Michael Ruf, George N. Harakas

**Affiliations:** a PO Box 6949, Radford University, Radford, Virginia 24142, USA; b Bruker AXS, 5465 E Cheryl Pkwy, Madison, Wisconsin 53711, USA; c Bruker AXS, 5465 E Cheryl Pkwy, Madison, Wisconson 53711, USA; Goethe-Universität Frankfurt, Germany

**Keywords:** crystal structure, organometallic, iron, gallium, siloxane, hydrogen bonding

## Abstract

The title compound, [Fe(C_4_H_8_O)_4_(H_2_O)_2_][Fe_4_Ga_4_(C_2_H_6_O_2_Si)Cl_4_(CO)_15_]·4C_4_H_8_O, consists of an iron(II) cation octa­hedrally coordinated by two water mol­ecules (*trans*) with four tetra­hydro­furans (THF) at equatorial sites. Two additional THF mol­ecules are hydrogen bonded to each of the water mol­ecules. The dianion of the title compound is an organometallic butterfly complex with a dimethyl siloxane core and two iron-gallium fragments. The lengths of the iron to gallium metal–metal bonds range from 2.3875 (6) to 2.4912 (6) Å.

## Abstract 2

The title compound consists of an octa­hedral iron(II) cation coordinated to two water mol­ecules (*trans*) with four tetra­hydro­furan (THF) ligands. Two additional THF mol­ecules are hydrogen bonded to each of the water mol­ecules. The dianion of the title compound is an organometallic butterfly complex with a di­methyl­siloxane core and two iron-gallium fragments.

## Graphical abstract

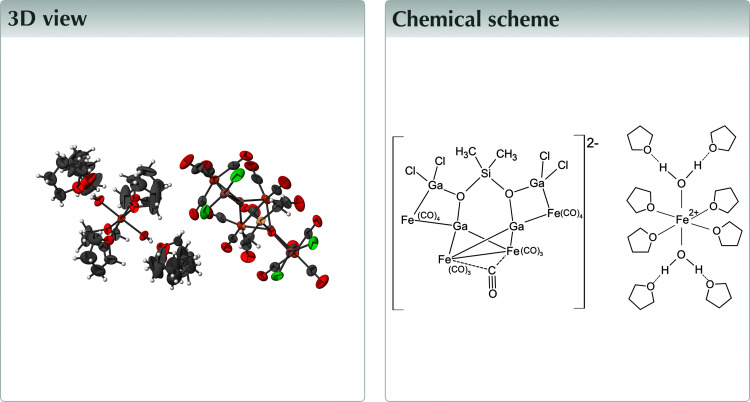

## Structure description

The accidental incorporation of silicone vacuum grease into chemical reactions has produced several unique compounds that contain di­methyl­siloxane fragments (Haiduc, 2004 ▸). In our study of the chemical reactivity of digallium(II) dichloride with transition-metal clusters we now report the formation of the title compound, which contains a di­methyl­siloxane-gallium-iron butterfly dianion cluster (Li & Rauchfuss, 2016 ▸). The Ga_2_Cl_4_ solution, see *Synthesis and crystallization* section, used in this reaction was contaminated with silicone vacuum grease. This inter­action produced a gallium-siloxane inter­mediate that has yet to be identified. The vacuum grease is also thought to be the source of water that is observed coordinating to the iron(II) cation.

**Figure 1:**
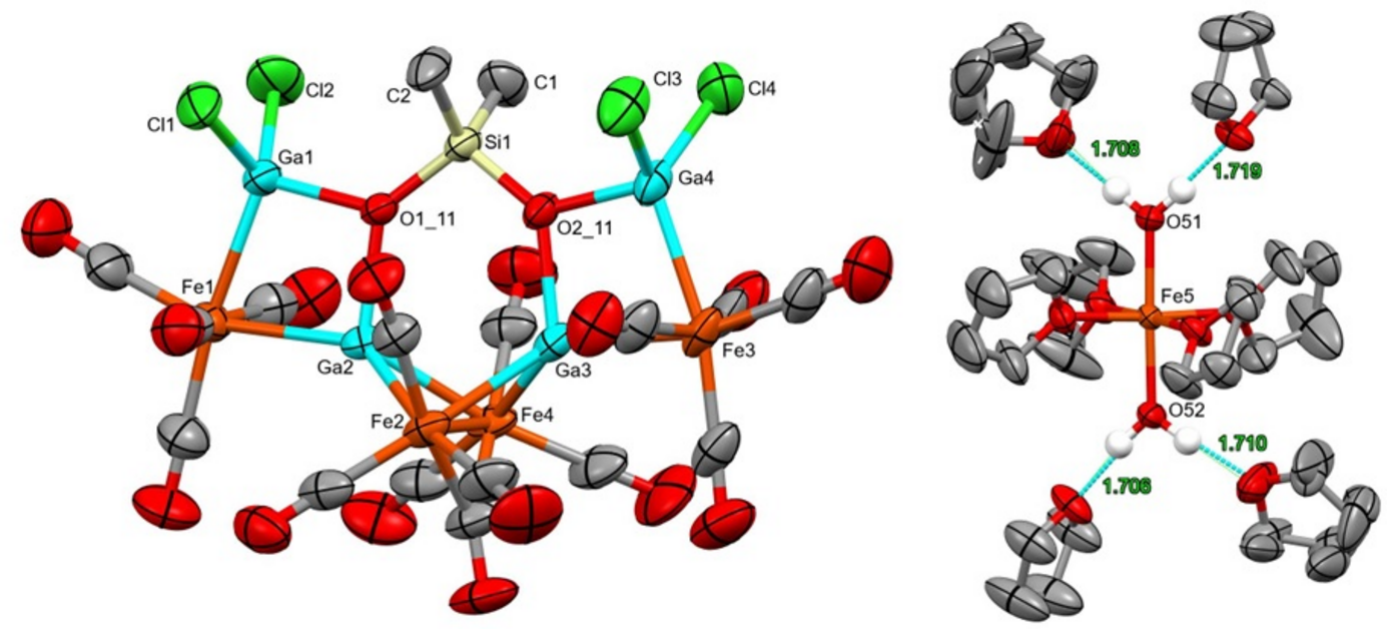
The mol­ecular structures within the title compound, with displacement ellipsoids drawn at the 50% probability level. Note, this is a composite image of the cation and anion, which were rendered separately for clarity. The hydrogen atoms have been omitted with the exception of the two water mol­ecules participating in hydrogen bonding.

**Figure 2:**
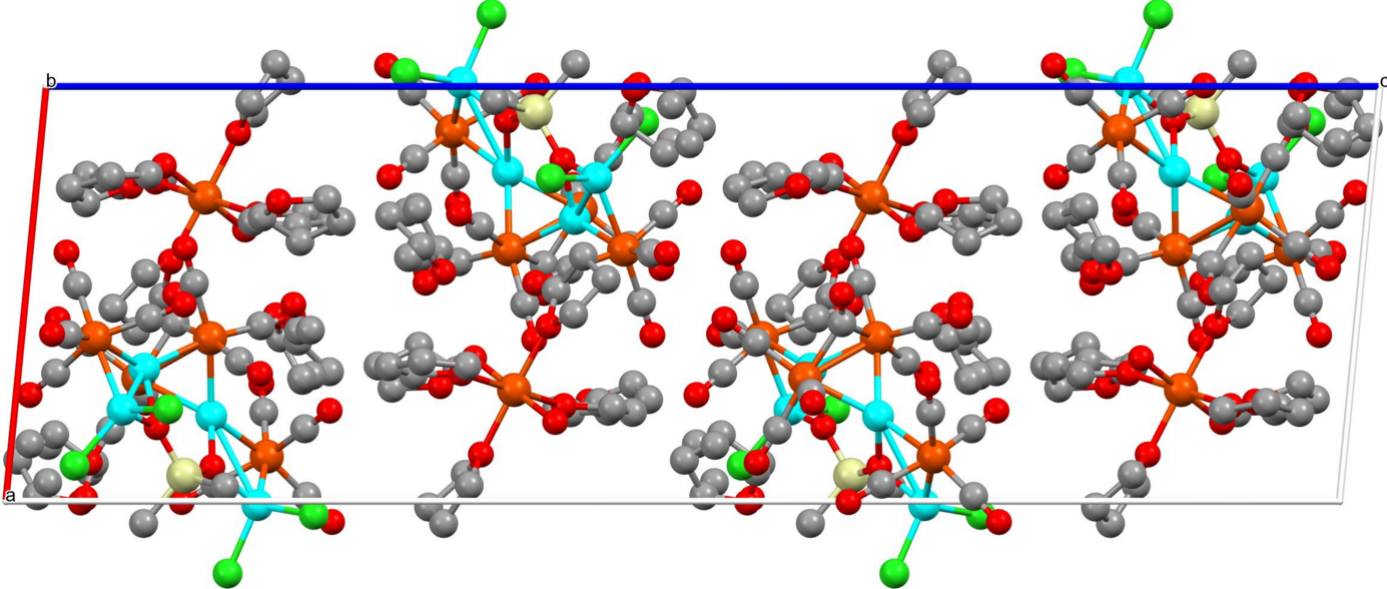
Crystal packing diagram viewed along the *b* axis; hydrogen atoms have been omitted for clarity.

The dianion of the title compound has a dimethyl siloxane entity in which the oxygen atoms are binding to the gallium atoms. There are two types of gallium atoms in this cluster: two belong to GaCl_2_ fragments and two gallium atoms are bound only to oxygen or iron atoms (Fig. 1 ▸). The distances between Ga1—Ga2 and Ga3—Ga4 are 2.9476 (5) Å and 2.9736 (6) Å, respectively. These values are much longer than 2.406 Å observed for Ga_2_Cl_4_·2(1,4-dioxane) (Beamish *et al.*, 1979 ▸), indicating there are no metal–metal bonding inter­actions between the pairs of gallium atoms. The iron–gallium and iron–iron bond lengths of the dianion are listed in Table 1 ▸. The dication of the title compound is an octa­hedral coordination complex, *trans*-[Fe(H_2_O)_2_(THF)_4_]^2+^. Each of the water mol­ecules is hydrogen bonded to two additional THF mol­ecules (Table 2 ▸) in the crystal structure (Fig. 2 ▸).

## Synthesis and crystallization

All manipulations were conducted using inert atmosphere techniques. A stock solution of Ga_2_Cl_4_ was produced by the reaction of Ga (5.496 g, 78.83 mmol) with GaCl_3_ (5.01 g, 28.4 mmol) in 150 ml of toluene. The mixture was heated to reflux for 24 h then cooled to 25°C. Note: after the title compound’s structure was determined, it was noted visually that a small amount of silicone vacuum grease had contaminated this stock solution.

In a 150 ml Schlenk flask, Fe_3_(CO)_12_ (0.493 g, 0.979 mmol) in 35 ml of toluene was combined with 10 ml of the Ga_2_Cl_4_ stock solution. The reaction flask was refluxed for 2 h. During this time the reaction mixture changed from a deep green/black color to dark orange. The mixture was cooled to room temperature, and the solution was deca­nted away from the residue into a new Schlenk flask, 20 ml of THF were added to this solution. The solution was cooled to −15°C and orange crystals were observed after 72 h. A single crystal was coated with NVH oil and mounted on a MiTeGen loop under a stream of argon gas then cooled to −70°C for data collection.

## Refinement

Crystal data, data collection, and structure refinement details are summarized in Table 3 ▸. The THF mol­ecules were refined using the SAME restraint. The water mol­ecules (O51, O52) bound to Fe5 were refined with the *DFIX* and SADI (same distances with standard deviation of 0.01 Å) restraints. Two THF mol­ecules hydrogen bonding to the water mol­ecules were split into residues 6 and 7 and residues 9 and 10, respectively, and modeled for disorder. The occupancy values of each residue pair were 48.6/51.4 and 48.4/51.6, respectively.

## Supplementary Material

Crystal structure: contains datablock(s) global, I. DOI: 10.1107/S2414314624006205/bt4151sup1.cif

Structure factors: contains datablock(s) I. DOI: 10.1107/S2414314624006205/bt4151Isup2.hkl

CCDC reference: 2365148

Additional supporting information:  crystallographic information; 3D view; checkCIF report

## Figures and Tables

**Table 1:** Selected bond lengths (Å)

Ga1—Fe1	2.3875 (6)	Ga3—Fe4	2.4301 (6)
Ga2—Fe4	2.4202 (6)	Ga3—Fe3	2.4786 (6)
Ga2—Fe2	2.4243 (5)	Ga4—Fe3	2.4010 (6)
Ga2—Fe1	2.4912 (6)	Fe2—Fe4	2.7027 (6)
Ga3—Fe2	2.4293 (6)		

**Table 2:** Hydrogen-bond geometry (Å, °)

*D*—H⋯*A*	*D*—H	H⋯*A*	*D*⋯*A*	*D*—H⋯*A*
O51—H51*A*⋯O1_5	0.96 (2)	1.72 (2)	2.677 (3)	175 (2)
O51—H51*B*⋯O1_7	0.96 (2)	1.71 (2)	2.623 (11)	159 (2)
O51—H51*B*⋯O1_6	0.96 (2)	1.71 (2)	2.671 (15)	179 (3)
O52—H52*A*⋯O1_8	0.96 (2)	1.71 (2)	2.653 (4)	170 (3)
O52—H52*B*⋯O1_10	0.96 (2)	1.71 (2)	2.665 (12)	175 (3)
O52—H52*B*⋯O1_9	0.96 (2)	1.71 (3)	2.647 (16)	167 (3)

**Table 3:** Experimental details

Crystal data	
Chemical formula	[Fe(C_4_H_8_O)_4_(H_2_O)_2_][Fe_4_Ga_4_(C_2_H_6_O_2_Si)Cl_4_(CO)_15_]·4C_4_H_8_O
*M*_r_	1823.10
Crystal system, space group	Monoclinic, *P*2_1_/*c*
Temperature (K)	203
*a*, *b*, *c* (Å)	12.1954 (6), 15.2657 (7), 39.0300 (16)
β (°)	95.5629 (16)
*V* (Å^3^)	7232.0 (6)
*Z*	4
Radiation type	Mo *K*α
μ (mm^−1^)	2.68
Crystal size (mm)	0.15 × 0.13 × 0.06

Data collection	
Diffractometer	Bruker D8 Quest Eco, Photon II 7
Absorption correction	Multi-scan (*SADABS*; Krause *et al.*, 2015 ▸)
*T*_min_, *T*_max_	0.65, 0.75
No. of measured, independent and observed [*I* > 2σ(*I*)] reflections	157023, 17970, 14164
*R*_int_	0.038
(sin θ/λ)_max_ (Å^−1^)	0.668

Refinement	
*R*[*F*^2^ > 2σ(*F*^2^)], *wR*(*F*^2^), *S*	0.041, 0.100, 1.04
No. of reflections	17970
No. of parameters	919
No. of restraints	471
H-atom treatment	H atoms treated by a mixture of independent and constrained refinement
Δρ_max_, Δρ_min_ (e Å^−3^)	0.86, −0.52

Computer programs: *APEX3* and *SAINT* (Bruker, 2019 ▸), *SHELXT2018/2* (Sheldrick, 2015*a* ▸), *SHELXL2018/3* (Sheldrick, 2015*b* ▸) and *ShelXle* (Hübschle *et al.*, 2011 ▸).

## full crystallographic data

### trans-Diaquatetrakis(tetrahydrofuran-κO)iron(II) µ-carbonyl-tetradecacarbonyltetrachlorido-µ-dimethylsilanediolato-tetragalliumtetrairon(7 Ga–Fe)(Fe–Fe) tetrahydrofuran tetrasolvate Crystal data

**Table:**

[Fe(C_4_H_8_O)_4_(H_2_O)_2_][Fe_4_Ga_4_(C_2_H_6_O_2_Si)Cl_4_(CO)_15_]·4C_4_H_8_O	*F*(000) = 3680
*M**_r_* = 1823.10	*D*_x_ = 1.674 Mg m^−^^3^
Monoclinic, *P*2_1_/*c*	Mo *K*α radiation, λ = 0.71073 Å
*a* = 12.1954 (6) Å	Cell parameters from 9631 reflections
*b* = 15.2657 (7) Å	θ = 2.5–27.8°
*c* = 39.0300 (16) Å	µ = 2.68 mm^−^^1^
β = 95.5629 (16)°	*T* = 203 K
*V* = 7232.0 (6) Å^3^	Block, orange
*Z* = 4	0.15 × 0.13 × 0.06 mm

### trans-Diaquatetrakis(tetrahydrofuran-κO)iron(II) µ-carbonyl-tetradecacarbonyltetrachlorido-µ-dimethylsilanediolato-tetragalliumtetrairon(7 Ga–Fe)(Fe–Fe) tetrahydrofuran tetrasolvate Data collection

**Table:**

Bruker D8 Quest Eco, Photon II 7 diffractometer	14164 reflections with *I* > 2σ(*I*)
Detector resolution: 7.3910 pixels mm^-1^	*R*_int_ = 0.038
phi and ω scans	θ_max_ = 28.3°, θ_min_ = 2.9°
Absorption correction: multi-scan (SADABS; Krause *et al.*, 2015)	*h* = −16→16
*T*_min_ = 0.65, *T*_max_ = 0.75	*k* = −20→20
157023 measured reflections	*l* = −52→52
17970 independent reflections	

### trans-Diaquatetrakis(tetrahydrofuran-κO)iron(II) µ-carbonyl-tetradecacarbonyltetrachlorido-µ-dimethylsilanediolato-tetragalliumtetrairon(7 Ga–Fe)(Fe–Fe) tetrahydrofuran tetrasolvate Refinement

**Table:**

Refinement on *F*^2^	471 restraints
Least-squares matrix: full	Hydrogen site location: mixed
*R*[*F*^2^ > 2σ(*F*^2^)] = 0.041	H atoms treated by a mixture of independent and constrained refinement
*wR*(*F*^2^) = 0.100	*w* = 1/[σ^2^(*F*_o_^2^) + (0.039*P*)^2^ + 9.8448*P*] where *P* = (*F*_o_^2^ + 2*F*_c_^2^)/3
*S* = 1.04	(Δ/σ)_max_ = 0.003
17970 reflections	Δρ_max_ = 0.86 e Å^−^^3^
919 parameters	Δρ_min_ = −0.52 e Å^−^^3^

### trans-Diaquatetrakis(tetrahydrofuran-κO)iron(II) µ-carbonyl-tetradecacarbonyltetrachlorido-µ-dimethylsilanediolato-tetragalliumtetrairon(7 Ga–Fe)(Fe–Fe) tetrahydrofuran tetrasolvate Special details

**Table:**

Geometry. All esds (except the esd in the dihedral angle between two l.s. planes) are estimated using the full covariance matrix. The cell esds are taken into account individually in the estimation of esds in distances, angles and torsion angles; correlations between esds in cell parameters are only used when they are defined by crystal symmetry. An approximate (isotropic) treatment of cell esds is used for estimating esds involving l.s. planes.

### trans-Diaquatetrakis(tetrahydrofuran-κO)iron(II) µ-carbonyl-tetradecacarbonyltetrachlorido-µ-dimethylsilanediolato-tetragalliumtetrairon(7 Ga–Fe)(Fe–Fe) tetrahydrofuran tetrasolvate Fractional atomic coordinates and isotropic or equivalent isotropic displacement parameters (Å^2^)

**Table:**

	*x*	*y*	*z*	*U*_iso_*/*U*_eq_	Occ. (<1)
Ga1	0.77957 (3)	0.40052 (2)	0.58089 (2)	0.04592 (9)	
Ga2	0.68040 (3)	0.22925 (2)	0.59417 (2)	0.03728 (8)	
Ga3	0.79472 (3)	0.09718 (2)	0.64778 (2)	0.04118 (8)	
Ga4	1.00953 (3)	0.12783 (2)	0.68890 (2)	0.04801 (9)	
Fe1	0.60664 (4)	0.34838 (3)	0.55484 (2)	0.04806 (12)	
Fe2	0.60697 (4)	0.15225 (3)	0.64124 (2)	0.04336 (11)	
Fe3	0.89249 (5)	0.00230 (3)	0.69235 (2)	0.05294 (13)	
Fe4	0.70437 (4)	0.07230 (3)	0.59034 (2)	0.04596 (12)	
Cl2	0.91426 (9)	0.42695 (9)	0.54905 (3)	0.0883 (4)	
Cl3	1.03304 (11)	0.22061 (7)	0.73190 (3)	0.0757 (3)	
Cl4	1.17461 (9)	0.11436 (7)	0.67154 (3)	0.0738 (3)	
Si1	0.93847 (7)	0.27812 (5)	0.63190 (2)	0.03962 (18)	
Fe5	0.26811 (3)	0.68605 (3)	0.62708 (2)	0.03695 (10)	
Cl1	0.77826 (10)	0.51465 (6)	0.61509 (3)	0.0793 (3)	
O51	0.34485 (19)	0.59098 (14)	0.65940 (6)	0.0482 (5)	
H51A	0.318 (2)	0.5355 (11)	0.6666 (8)	0.058 (11)*	
H51B	0.4193 (9)	0.594 (2)	0.6697 (6)	0.082 (14)*	
O52	0.19090 (19)	0.77799 (15)	0.59388 (7)	0.0532 (6)	
C1	1.0537 (3)	0.2641 (3)	0.60489 (11)	0.0657 (11)	
H1A	1.069947	0.320358	0.594369	0.099000*	
H1B	1.033151	0.221062	0.586778	0.099000*	
H1C	1.119094	0.243551	0.619206	0.099000*	
C2	0.9661 (4)	0.3679 (2)	0.66305 (10)	0.0647 (11)	
H2A	0.970605	0.423386	0.650663	0.097000*	
H2B	1.035988	0.356934	0.676979	0.097000*	
H2C	0.906348	0.371037	0.678088	0.097000*	
H52A	0.213 (3)	0.8359 (10)	0.5881 (8)	0.078000*	
H52B	0.1181 (12)	0.771 (2)	0.5826 (7)	0.078000*	
O1_1	0.2295 (2)	0.58691 (14)	0.58859 (6)	0.0506 (6)	
C1_1	0.2257 (5)	0.6008 (3)	0.55217 (10)	0.0894 (16)	
H1A_1	0.294257	0.629295	0.546226	0.107000*	
H1B_1	0.162362	0.638508	0.544116	0.107000*	
C2_1	0.2140 (9)	0.5155 (4)	0.53664 (14)	0.173 (4)	
H2A_1	0.137893	0.507920	0.525741	0.208000*	
H2B_1	0.265172	0.509773	0.518553	0.208000*	
C3_1	0.2379 (5)	0.4500 (3)	0.56229 (12)	0.0845 (14)	
H3A_1	0.315218	0.429823	0.562805	0.101000*	
H3B_1	0.188116	0.398980	0.558313	0.101000*	
C4_1	0.2186 (6)	0.4957 (3)	0.59413 (11)	0.101 (2)	
H4A_1	0.143804	0.482409	0.600503	0.121000*	
H4B_1	0.272865	0.476340	0.613139	0.121000*	
O1_2	0.11247 (19)	0.67109 (14)	0.64724 (7)	0.0509 (6)	
C1_2	0.0384 (4)	0.7421 (3)	0.65381 (14)	0.0774 (13)	
H1A_2	0.011183	0.771229	0.631971	0.093000*	
H1B_2	0.077119	0.786094	0.669206	0.093000*	
C2_2	−0.0537 (4)	0.7037 (3)	0.67018 (12)	0.0699 (11)	
H2A_2	−0.119648	0.698092	0.653398	0.084000*	
H2B_2	−0.072662	0.740411	0.689661	0.084000*	
C3_2	−0.0142 (3)	0.6158 (2)	0.68256 (10)	0.0613 (10)	
H3A_2	0.022860	0.619034	0.706235	0.074000*	
H3B_2	−0.075841	0.573405	0.682095	0.074000*	
C4_2	0.0645 (3)	0.5911 (2)	0.65752 (12)	0.0644 (11)	
H4A_2	0.122039	0.551495	0.668367	0.077000*	
H4B_2	0.025728	0.561079	0.637383	0.077000*	
O1_3	0.3085 (2)	0.77824 (15)	0.66755 (6)	0.0536 (6)	
C1_3	0.3265 (5)	0.8703 (3)	0.66421 (13)	0.0911 (16)	
H1A_3	0.263766	0.897553	0.650108	0.109000*	
H1B_3	0.394474	0.881069	0.652876	0.109000*	
C2_3	0.3370 (7)	0.9069 (4)	0.69824 (15)	0.128 (3)	
H2A_3	0.410279	0.934671	0.702991	0.154000*	
H2B_3	0.280088	0.952499	0.699999	0.154000*	
C3_3	0.3244 (7)	0.8401 (4)	0.72266 (14)	0.116 (2)	
H3A_3	0.261318	0.853389	0.735954	0.139000*	
H3B_3	0.391728	0.835410	0.738900	0.139000*	
C4_3	0.3051 (5)	0.7581 (3)	0.70368 (10)	0.0829 (14)	
H4A_3	0.362617	0.714627	0.711275	0.099000*	
H4B_3	0.232325	0.733475	0.707726	0.099000*	
O1_4	0.42211 (19)	0.70737 (15)	0.60597 (7)	0.0531 (6)	
C1_4	0.5081 (4)	0.6456 (3)	0.60289 (19)	0.110 (2)	
H1A_4	0.480296	0.593961	0.589349	0.133000*	
H1B_4	0.540106	0.625686	0.625851	0.133000*	
C2_4	0.5903 (6)	0.6936 (5)	0.5849 (3)	0.217 (6)	
H2A_4	0.648189	0.718019	0.601808	0.260000*	
H2B_4	0.625642	0.653791	0.569263	0.260000*	
C3_4	0.5347 (5)	0.7614 (4)	0.56616 (15)	0.1048 (19)	
H3A_4	0.502622	0.740899	0.543286	0.126000*	
H3B_4	0.583984	0.811799	0.563172	0.126000*	
C4_4	0.4508 (6)	0.7831 (4)	0.5880 (2)	0.168 (4)	
H4A_4	0.478028	0.829088	0.604591	0.201000*	
H4B_4	0.385076	0.806115	0.573964	0.201000*	
O1_5	0.2774 (2)	0.43712 (15)	0.68269 (6)	0.0570 (6)	
C1_5	0.2724 (4)	0.4325 (3)	0.71875 (10)	0.0734 (12)	
H1A_5	0.194733	0.433082	0.724138	0.088000*	
H1B_5	0.310705	0.483414	0.730177	0.088000*	
C2_5	0.3261 (6)	0.3509 (4)	0.73089 (12)	0.106 (2)	
H2A_5	0.384336	0.362976	0.749772	0.127000*	
H2B_5	0.271653	0.310418	0.739563	0.127000*	
C3_5	0.3738 (4)	0.3122 (3)	0.70159 (11)	0.0790 (13)	
H3A_5	0.362096	0.248056	0.700858	0.095000*	
H3B_5	0.453791	0.324320	0.702726	0.095000*	
C4_5	0.3150 (4)	0.3547 (2)	0.67147 (10)	0.0643 (10)	
H4A_5	0.364952	0.362976	0.653191	0.077000*	
H4B_5	0.251896	0.318213	0.662173	0.077000*	
O1_6	0.5518 (10)	0.5996 (9)	0.6887 (6)	0.116 (7)	0.49 (2)
C1_6	0.6063 (18)	0.5196 (10)	0.6939 (7)	0.153 (12)	0.49 (2)
H1A_6	0.618835	0.492660	0.671514	0.184000*	0.49 (2)
H1B_6	0.561776	0.478748	0.706559	0.184000*	0.49 (2)
C2_6	0.7088 (11)	0.5374 (11)	0.7134 (5)	0.090 (6)	0.49 (2)
H2A_6	0.767292	0.548791	0.698065	0.108000*	0.49 (2)
H2B_6	0.731537	0.487634	0.728732	0.108000*	0.49 (2)
C3_6	0.688 (2)	0.6143 (15)	0.7331 (5)	0.153 (12)	0.49 (2)
H3A_6	0.756149	0.647302	0.740068	0.184000*	0.49 (2)
H3B_6	0.650236	0.599680	0.753772	0.184000*	0.49 (2)
C4_6	0.614 (2)	0.6623 (8)	0.7072 (7)	0.24 (2)	0.49 (2)
H4A_6	0.566391	0.703173	0.718599	0.292000*	0.49 (2)
H4B_6	0.658263	0.696424	0.691798	0.292000*	0.49 (2)
O1_7	0.5265 (8)	0.5944 (8)	0.7013 (3)	0.071 (3)	0.51 (2)
C1_7	0.5886 (11)	0.5193 (6)	0.7071 (5)	0.085 (5)	0.51 (2)
H1A_7	0.591833	0.486304	0.685422	0.102000*	0.51 (2)
H1B_7	0.555783	0.481053	0.723912	0.102000*	0.51 (2)
C2_7	0.6970 (13)	0.5465 (11)	0.7205 (7)	0.135 (11)	0.51 (2)
H2A_7	0.753719	0.513401	0.709404	0.162000*	0.51 (2)
H2B_7	0.708271	0.536484	0.745683	0.162000*	0.51 (2)
C3_7	0.7038 (10)	0.6385 (9)	0.7129 (6)	0.113 (7)	0.51 (2)
H3A_7	0.725255	0.648959	0.689421	0.136000*	0.51 (2)
H3B_7	0.755151	0.669629	0.729928	0.136000*	0.51 (2)
C4_7	0.5875 (11)	0.6627 (11)	0.7159 (6)	0.193 (14)	0.51 (2)
H4A_7	0.574103	0.670133	0.740374	0.232000*	0.51 (2)
H4B_7	0.568537	0.718069	0.703500	0.232000*	0.51 (2)
O1_8	0.2402 (3)	0.93500 (16)	0.57058 (8)	0.0738 (8)	
C1_8	0.2805 (6)	0.9465 (3)	0.53812 (13)	0.106 (2)	
H1A_8	0.250871	0.900473	0.521930	0.128000*	
H1B_8	0.361969	0.942784	0.540390	0.128000*	
C2_8	0.2450 (7)	1.0325 (4)	0.52549 (15)	0.125 (3)	
H2A_8	0.305105	1.062485	0.514811	0.150000*	
H2B_8	0.180427	1.027367	0.508166	0.150000*	
C3_8	0.2168 (7)	1.0795 (3)	0.55489 (15)	0.132 (3)	
H3A_8	0.144215	1.108166	0.549842	0.158000*	
H3B_8	0.272525	1.125445	0.561170	0.158000*	
C4_8	0.2130 (5)	1.0178 (3)	0.58307 (13)	0.0936 (17)	
H4A_8	0.266317	1.034944	0.602677	0.112000*	
H4B_8	0.138347	1.016605	0.591010	0.112000*	
O1_9	0.0034 (12)	0.7454 (16)	0.5560 (4)	0.135 (10)	0.48 (2)
C1_9	−0.0130 (15)	0.7815 (14)	0.5230 (5)	0.102 (8)	0.48 (2)
H1A_9	0.055067	0.778042	0.511205	0.123000*	0.48 (2)
H1B_9	−0.036108	0.843574	0.524052	0.123000*	0.48 (2)
C2_9	−0.0987 (14)	0.7289 (14)	0.5058 (3)	0.103 (7)	0.48 (2)
H2A_9	−0.066565	0.681985	0.492429	0.123000*	0.48 (2)
H2B_9	−0.147241	0.765433	0.489797	0.123000*	0.48 (2)
C3_9	−0.160 (2)	0.692 (2)	0.5316 (5)	0.140 (13)	0.48 (2)
H3A_9	−0.179678	0.629910	0.525992	0.168000*	0.48 (2)
H3B_9	−0.229350	0.724934	0.533372	0.168000*	0.48 (2)
C4_9	−0.0904 (11)	0.6966 (10)	0.5635 (2)	0.066 (4)	0.48 (2)
H4A_9	−0.128906	0.726350	0.581435	0.080000*	0.48 (2)
H4B_9	−0.068544	0.637199	0.571792	0.080000*	0.48 (2)
O1_10	−0.0063 (9)	0.7554 (11)	0.5592 (3)	0.074 (4)	0.52 (2)
C1_10	−0.0446 (16)	0.8027 (12)	0.5299 (6)	0.123 (9)	0.52 (2)
H1A_10	0.013766	0.807664	0.514109	0.148000*	0.52 (2)
H1B_10	−0.066544	0.862472	0.536422	0.148000*	0.52 (2)
C2_10	−0.138 (2)	0.7563 (13)	0.5136 (7)	0.166 (12)	0.52 (2)
H2A_10	−0.134177	0.754275	0.488358	0.200000*	0.52 (2)
H2B_10	−0.207195	0.785895	0.518197	0.200000*	0.52 (2)
C3_10	−0.1346 (19)	0.6690 (11)	0.5276 (5)	0.105 (8)	0.52 (2)
H3A_10	−0.209824	0.647464	0.530313	0.126000*	0.52 (2)
H3B_10	−0.098524	0.628085	0.512507	0.126000*	0.52 (2)
C4_10	−0.071 (2)	0.6776 (16)	0.5604 (6)	0.216 (16)	0.52 (2)
H4A_10	−0.121101	0.682088	0.578926	0.259000*	0.52 (2)
H4B_10	−0.023357	0.625809	0.564948	0.259000*	0.52 (2)
O1_11	0.82171 (16)	0.29850 (12)	0.60764 (5)	0.0362 (4)	
O2_11	0.91756 (17)	0.18584 (13)	0.65235 (5)	0.0382 (4)	
O1_12	1.0022 (3)	−0.04848 (18)	0.63186 (8)	0.0753 (8)	
O2_12	1.0503 (3)	−0.0712 (3)	0.74608 (10)	0.1052 (13)	
O3_12	0.7166 (3)	−0.1293 (2)	0.68296 (10)	0.0918 (11)	
O4_12	0.7819 (3)	0.1230 (2)	0.73612 (7)	0.0766 (9)	
O5_12	0.7440 (3)	0.2532 (2)	0.51010 (8)	0.0814 (9)	
O6_12	0.5970 (3)	0.5219 (2)	0.52254 (10)	0.1065 (13)	
O7_12	0.4004 (3)	0.2637 (3)	0.52738 (10)	0.0952 (11)	
O8_12	0.5260 (2)	0.40701 (19)	0.61909 (8)	0.0686 (7)	
C1_12	0.9590 (3)	−0.0278 (2)	0.65536 (11)	0.0586 (9)	
C2_12	0.9884 (4)	−0.0446 (3)	0.72526 (12)	0.0704 (11)	
C3_12	0.7856 (4)	−0.0789 (2)	0.68688 (11)	0.0668 (11)	
C4_12	0.8254 (4)	0.0764 (3)	0.71876 (10)	0.0611 (10)	
C5_12	0.6913 (4)	0.2903 (3)	0.52803 (10)	0.0612 (10)	
C6_12	0.5982 (4)	0.4535 (3)	0.53427 (11)	0.0719 (12)	
C7_12	0.4798 (3)	0.2957 (3)	0.53794 (11)	0.0665 (10)	
C8_12	0.5588 (3)	0.3828 (2)	0.59448 (11)	0.0530 (8)	
O1_13	0.5019 (3)	−0.00811 (19)	0.61183 (9)	0.0805 (9)	
O2_13	0.6932 (3)	0.30366 (18)	0.68131 (7)	0.0671 (7)	
O3_13	0.3895 (2)	0.2195 (2)	0.61564 (9)	0.0763 (9)	
O4_13	0.5540 (3)	0.0537 (3)	0.70176 (9)	0.1019 (12)	
O5_13	0.9073 (3)	0.1146 (2)	0.56012 (9)	0.0860 (10)	
O6_13	0.5616 (3)	0.0767 (3)	0.52553 (8)	0.0927 (11)	
O7_13	0.7630 (4)	−0.11239 (19)	0.59787 (12)	0.1096 (13)	
C1_13	0.5697 (3)	0.0463 (2)	0.61383 (10)	0.0561 (9)	
C2_13	0.6626 (3)	0.2446 (2)	0.66472 (9)	0.0485 (8)	
C3_13	0.4747 (3)	0.1934 (2)	0.62536 (10)	0.0558 (9)	
C4_13	0.5770 (4)	0.0912 (3)	0.67814 (11)	0.0670 (11)	
C5_13	0.8286 (3)	0.1022 (3)	0.57329 (10)	0.0584 (9)	
C6_13	0.6168 (3)	0.0757 (3)	0.55091 (11)	0.0644 (11)	
C7_13	0.7396 (4)	−0.0403 (3)	0.59586 (12)	0.0700 (12)	

### trans-Diaquatetrakis(tetrahydrofuran-κO)iron(II) µ-carbonyl-tetradecacarbonyltetrachlorido-µ-dimethylsilanediolato-tetragalliumtetrairon(7 Ga–Fe)(Fe–Fe) tetrahydrofuran tetrasolvate Atomic displacement parameters (Å^2^)

**Table:**

	*U*^11^	*U*^22^	*U*^33^	*U*^12^	*U*^13^	*U*^23^
Ga1	0.04209 (19)	0.03764 (18)	0.0556 (2)	−0.00983 (14)	−0.00741 (15)	0.01288 (15)
Ga2	0.03962 (17)	0.03366 (16)	0.03902 (16)	−0.00954 (13)	0.00611 (13)	−0.00433 (13)
Ga3	0.0520 (2)	0.02605 (15)	0.04740 (19)	−0.00551 (14)	0.01476 (15)	−0.00005 (13)
Ga4	0.0610 (2)	0.03284 (17)	0.0497 (2)	0.00230 (16)	0.00277 (17)	0.01046 (15)
Fe1	0.0433 (3)	0.0511 (3)	0.0474 (3)	−0.0075 (2)	−0.0080 (2)	0.0034 (2)
Fe2	0.0475 (3)	0.0345 (2)	0.0510 (3)	−0.01122 (19)	0.0198 (2)	−0.00626 (19)
Fe3	0.0738 (3)	0.0292 (2)	0.0587 (3)	0.0017 (2)	0.0210 (3)	0.0125 (2)
Fe4	0.0505 (3)	0.0346 (2)	0.0548 (3)	−0.01308 (19)	0.0154 (2)	−0.0167 (2)
Cl2	0.0629 (6)	0.1043 (9)	0.0984 (8)	−0.0179 (6)	0.0120 (6)	0.0548 (7)
Cl3	0.1146 (9)	0.0591 (6)	0.0491 (5)	−0.0039 (6)	−0.0133 (5)	0.0016 (4)
Cl4	0.0568 (6)	0.0654 (6)	0.0993 (8)	0.0085 (5)	0.0073 (5)	0.0160 (6)
Si1	0.0421 (4)	0.0305 (4)	0.0447 (4)	−0.0093 (3)	−0.0039 (3)	0.0082 (3)
Fe5	0.0377 (2)	0.0287 (2)	0.0433 (2)	−0.00448 (16)	−0.00214 (17)	0.00509 (17)
Cl1	0.0753 (7)	0.0368 (5)	0.1177 (9)	0.0003 (4)	−0.0315 (6)	−0.0114 (5)
O51	0.0491 (13)	0.0356 (11)	0.0568 (13)	−0.0027 (10)	−0.0113 (11)	0.0099 (10)
O52	0.0489 (13)	0.0391 (12)	0.0683 (15)	−0.0100 (10)	−0.0106 (11)	0.0208 (11)
C1	0.046 (2)	0.080 (3)	0.072 (2)	−0.0053 (19)	0.0082 (18)	0.029 (2)
C2	0.082 (3)	0.0345 (17)	0.070 (2)	−0.0132 (17)	−0.032 (2)	0.0021 (16)
O1_1	0.0686 (15)	0.0398 (12)	0.0417 (12)	−0.0095 (11)	−0.0037 (11)	0.0015 (9)
C1_1	0.134 (5)	0.082 (3)	0.049 (2)	−0.015 (3)	−0.010 (3)	0.007 (2)
C2_1	0.367 (14)	0.098 (5)	0.060 (3)	−0.043 (7)	0.049 (5)	−0.022 (3)
C3_1	0.105 (4)	0.061 (3)	0.089 (3)	−0.002 (3)	0.014 (3)	−0.024 (2)
C4_1	0.205 (7)	0.043 (2)	0.054 (2)	−0.028 (3)	0.005 (3)	−0.0051 (18)
O1_2	0.0469 (13)	0.0295 (11)	0.0782 (16)	−0.0015 (9)	0.0149 (11)	0.0068 (10)
C1_2	0.070 (3)	0.040 (2)	0.125 (4)	0.0116 (19)	0.029 (3)	0.010 (2)
C2_2	0.064 (3)	0.062 (2)	0.088 (3)	0.010 (2)	0.023 (2)	0.001 (2)
C3_2	0.075 (3)	0.053 (2)	0.058 (2)	−0.0063 (19)	0.0192 (19)	−0.0008 (17)
C4_2	0.058 (2)	0.0364 (18)	0.103 (3)	−0.0052 (16)	0.026 (2)	0.0166 (19)
O1_3	0.0694 (16)	0.0362 (12)	0.0540 (14)	−0.0120 (11)	−0.0002 (11)	−0.0023 (10)
C1_3	0.138 (5)	0.048 (2)	0.086 (3)	−0.036 (3)	0.007 (3)	−0.003 (2)
C2_3	0.219 (8)	0.062 (3)	0.098 (4)	−0.033 (4)	−0.011 (5)	−0.022 (3)
C3_3	0.190 (7)	0.078 (4)	0.078 (3)	0.000 (4)	0.006 (4)	−0.030 (3)
C4_3	0.135 (5)	0.062 (3)	0.050 (2)	−0.006 (3)	0.002 (2)	−0.0050 (19)
O1_4	0.0438 (13)	0.0432 (13)	0.0735 (16)	−0.0046 (10)	0.0114 (11)	0.0091 (11)
C1_4	0.074 (3)	0.080 (3)	0.186 (6)	0.026 (3)	0.056 (4)	0.052 (4)
C2_4	0.142 (6)	0.127 (6)	0.412 (15)	0.065 (5)	0.182 (9)	0.147 (8)
C3_4	0.099 (4)	0.126 (5)	0.093 (4)	−0.038 (4)	0.031 (3)	0.021 (3)
C4_4	0.120 (5)	0.083 (4)	0.323 (11)	0.035 (4)	0.139 (7)	0.105 (6)
O1_5	0.0826 (18)	0.0356 (12)	0.0550 (14)	0.0090 (12)	0.0180 (12)	0.0066 (10)
C1_5	0.106 (4)	0.061 (2)	0.056 (2)	0.017 (2)	0.024 (2)	−0.0015 (19)
C2_5	0.154 (6)	0.105 (4)	0.060 (3)	0.042 (4)	0.018 (3)	0.024 (3)
C3_5	0.099 (4)	0.061 (3)	0.076 (3)	0.025 (2)	0.003 (3)	0.002 (2)
C4_5	0.096 (3)	0.043 (2)	0.055 (2)	0.013 (2)	0.014 (2)	−0.0007 (16)
O1_6	0.089 (8)	0.072 (6)	0.170 (16)	0.003 (6)	−0.068 (9)	0.019 (8)
C1_6	0.134 (17)	0.135 (17)	0.17 (2)	0.061 (13)	−0.075 (14)	−0.091 (15)
C2_6	0.047 (7)	0.094 (11)	0.130 (14)	−0.002 (7)	0.015 (8)	−0.012 (10)
C3_6	0.17 (2)	0.130 (19)	0.143 (17)	0.023 (15)	−0.091 (16)	−0.055 (14)
C4_6	0.114 (16)	0.041 (7)	0.54 (6)	−0.019 (8)	−0.14 (2)	−0.017 (15)
O1_7	0.054 (4)	0.070 (6)	0.082 (5)	−0.011 (3)	−0.028 (4)	−0.018 (4)
C1_7	0.071 (7)	0.044 (6)	0.130 (12)	−0.011 (5)	−0.034 (7)	0.034 (7)
C2_7	0.096 (14)	0.17 (2)	0.133 (17)	0.040 (14)	−0.022 (12)	0.081 (16)
C3_7	0.052 (7)	0.108 (11)	0.175 (18)	−0.020 (6)	−0.010 (9)	−0.008 (11)
C4_7	0.073 (9)	0.23 (2)	0.27 (2)	0.034 (11)	−0.045 (11)	−0.20 (2)
O1_8	0.109 (2)	0.0373 (13)	0.0802 (19)	−0.0065 (14)	0.0343 (17)	0.0116 (13)
C1_8	0.187 (6)	0.058 (3)	0.082 (3)	0.015 (3)	0.054 (4)	0.009 (2)
C2_8	0.205 (8)	0.081 (4)	0.094 (4)	0.041 (4)	0.050 (5)	0.030 (3)
C3_8	0.243 (9)	0.058 (3)	0.105 (4)	0.042 (4)	0.066 (5)	0.022 (3)
C4_8	0.136 (5)	0.061 (3)	0.092 (3)	0.014 (3)	0.053 (3)	0.009 (3)
O1_9	0.077 (10)	0.142 (17)	0.171 (19)	−0.059 (10)	−0.072 (11)	0.080 (13)
C1_9	0.051 (8)	0.143 (18)	0.110 (10)	−0.013 (9)	−0.012 (7)	0.077 (11)
C2_9	0.122 (15)	0.135 (16)	0.047 (6)	0.017 (10)	−0.013 (7)	−0.001 (7)
C3_9	0.097 (12)	0.20 (3)	0.119 (16)	−0.092 (17)	−0.019 (11)	0.000 (17)
C4_9	0.072 (7)	0.087 (8)	0.042 (5)	−0.024 (6)	0.015 (5)	−0.001 (5)
O1_10	0.063 (7)	0.067 (7)	0.087 (8)	−0.018 (5)	−0.022 (6)	0.023 (6)
C1_10	0.073 (11)	0.077 (8)	0.21 (2)	−0.019 (8)	−0.054 (11)	0.082 (10)
C2_10	0.107 (14)	0.124 (15)	0.24 (3)	−0.049 (13)	−0.096 (15)	0.096 (16)
C3_10	0.148 (18)	0.073 (8)	0.085 (11)	−0.028 (9)	−0.034 (10)	0.014 (7)
C4_10	0.154 (18)	0.149 (17)	0.31 (3)	−0.118 (15)	−0.134 (19)	0.137 (19)
O1_11	0.0378 (10)	0.0293 (10)	0.0407 (10)	−0.0088 (8)	−0.0005 (8)	0.0044 (8)
O2_11	0.0460 (12)	0.0270 (9)	0.0413 (11)	−0.0036 (8)	0.0018 (9)	0.0045 (8)
O1_12	0.095 (2)	0.0480 (15)	0.088 (2)	0.0109 (15)	0.0382 (18)	−0.0017 (14)
O2_12	0.114 (3)	0.086 (3)	0.112 (3)	0.006 (2)	−0.005 (2)	0.049 (2)
O3_12	0.101 (3)	0.0608 (19)	0.118 (3)	−0.0283 (18)	0.034 (2)	0.0041 (18)
O4_12	0.108 (2)	0.0662 (18)	0.0603 (16)	0.0095 (17)	0.0331 (16)	−0.0013 (14)
O5_12	0.107 (3)	0.085 (2)	0.0565 (17)	−0.0089 (19)	0.0285 (17)	−0.0046 (15)
O6_12	0.105 (3)	0.079 (2)	0.125 (3)	−0.012 (2)	−0.042 (2)	0.049 (2)
O7_12	0.0637 (19)	0.110 (3)	0.105 (3)	−0.0261 (19)	−0.0254 (18)	−0.015 (2)
O8_12	0.0659 (18)	0.0594 (17)	0.083 (2)	0.0030 (14)	0.0169 (15)	−0.0135 (15)
C1_12	0.078 (3)	0.0260 (15)	0.075 (2)	0.0041 (16)	0.022 (2)	0.0067 (16)
C2_12	0.089 (3)	0.047 (2)	0.077 (3)	0.002 (2)	0.017 (2)	0.024 (2)
C3_12	0.090 (3)	0.0396 (19)	0.075 (3)	−0.001 (2)	0.030 (2)	0.0119 (18)
C4_12	0.084 (3)	0.047 (2)	0.055 (2)	−0.0010 (19)	0.020 (2)	0.0133 (17)
C5_12	0.072 (3)	0.067 (2)	0.0436 (19)	−0.015 (2)	−0.0007 (18)	0.0062 (18)
C6_12	0.064 (3)	0.073 (3)	0.073 (3)	−0.012 (2)	−0.025 (2)	0.019 (2)
C7_12	0.058 (2)	0.072 (3)	0.066 (2)	−0.009 (2)	−0.0116 (19)	−0.003 (2)
C8_12	0.0446 (19)	0.0419 (18)	0.071 (2)	−0.0026 (14)	−0.0030 (17)	−0.0001 (17)
O1_13	0.0748 (19)	0.0574 (17)	0.114 (2)	−0.0376 (15)	0.0339 (18)	−0.0253 (16)
O2_13	0.087 (2)	0.0518 (15)	0.0625 (16)	−0.0082 (14)	0.0086 (14)	−0.0222 (13)
O3_13	0.0483 (16)	0.077 (2)	0.104 (2)	0.0003 (14)	0.0097 (15)	−0.0256 (17)
O4_13	0.109 (3)	0.113 (3)	0.091 (2)	−0.029 (2)	0.047 (2)	0.030 (2)
O5_13	0.0657 (19)	0.101 (2)	0.098 (2)	−0.0140 (17)	0.0410 (18)	−0.0178 (19)
O6_13	0.085 (2)	0.125 (3)	0.0654 (19)	−0.024 (2)	−0.0038 (17)	−0.035 (2)
O7_13	0.132 (3)	0.0340 (16)	0.164 (4)	−0.0034 (18)	0.022 (3)	−0.0297 (19)
C1_13	0.057 (2)	0.0412 (18)	0.073 (2)	−0.0153 (16)	0.0209 (18)	−0.0128 (16)
C2_13	0.055 (2)	0.0461 (18)	0.0469 (17)	−0.0035 (15)	0.0150 (15)	−0.0031 (15)
C3_13	0.052 (2)	0.050 (2)	0.069 (2)	−0.0139 (17)	0.0227 (18)	−0.0176 (17)
C4_13	0.067 (3)	0.064 (2)	0.075 (3)	−0.018 (2)	0.032 (2)	0.002 (2)
C5_13	0.058 (2)	0.055 (2)	0.065 (2)	−0.0100 (17)	0.0186 (18)	−0.0189 (18)
C6_13	0.065 (2)	0.067 (3)	0.063 (2)	−0.022 (2)	0.016 (2)	−0.029 (2)
C7_13	0.077 (3)	0.041 (2)	0.095 (3)	−0.0147 (19)	0.024 (2)	−0.027 (2)

### trans-Diaquatetrakis(tetrahydrofuran-κO)iron(II) µ-carbonyl-tetradecacarbonyltetrachlorido-µ-dimethylsilanediolato-tetragalliumtetrairon(7 Ga–Fe)(Fe–Fe) tetrahydrofuran tetrasolvate Geometric parameters (Å, º)

**Table:**

Ga1—O1_11	1.9172 (19)	C3_2—C4_2	1.483 (5)
Ga1—Cl2	2.1912 (11)	O1_3—C1_3	1.431 (4)
Ga1—Cl1	2.1958 (11)	O1_3—C4_3	1.448 (4)
Ga1—Fe1	2.3875 (6)	C1_3—C2_3	1.435 (6)
Ga1—Ga2	2.9476 (5)	C2_3—C3_3	1.415 (7)
Ga2—O1_11	2.0465 (19)	C3_3—C4_3	1.462 (6)
Ga2—Fe4	2.4202 (6)	O1_4—C4_4	1.414 (5)
Ga2—Fe2	2.4243 (5)	O1_4—C1_4	1.424 (5)
Ga2—Fe1	2.4912 (6)	C1_4—C2_4	1.473 (7)
Ga3—O2_11	2.014 (2)	C2_4—C3_4	1.405 (8)
Ga3—Fe2	2.4293 (6)	C3_4—C4_4	1.434 (7)
Ga3—Fe4	2.4301 (6)	O1_5—C1_5	1.416 (4)
Ga3—Fe3	2.4786 (6)	O1_5—C4_5	1.423 (4)
Ga3—Ga4	2.9736 (6)	C1_5—C2_5	1.466 (6)
Ga4—O2_11	1.941 (2)	C2_5—C3_5	1.457 (6)
Ga4—Cl3	2.1935 (11)	C3_5—C4_5	1.467 (5)
Ga4—Cl4	2.1952 (12)	O1_6—C4_6	1.384 (11)
Ga4—Fe3	2.4010 (6)	O1_6—C1_6	1.396 (12)
Fe1—C5_12	1.777 (5)	C1_6—C2_6	1.426 (12)
Fe1—C8_12	1.785 (4)	C2_6—C3_6	1.440 (14)
Fe1—C6_12	1.793 (4)	C3_6—C4_6	1.479 (14)
Fe1—C7_12	1.810 (4)	C1_7—C2_7	1.435 (12)
Fe2—C2_13	1.780 (4)	C2_7—C3_7	1.439 (13)
Fe2—C4_13	1.783 (4)	C3_7—C4_7	1.482 (13)
Fe2—C3_13	1.785 (4)	O1_8—C4_8	1.406 (5)
Fe2—C1_13	1.968 (3)	O1_8—C1_8	1.414 (5)
Fe2—Fe4	2.7027 (6)	C1_8—C2_8	1.453 (6)
Fe3—C4_12	1.782 (4)	C2_8—C3_8	1.424 (7)
Fe3—C1_12	1.783 (4)	C3_8—C4_8	1.453 (6)
Fe3—C3_12	1.796 (5)	O1_9—C1_9	1.396 (11)
Fe3—C2_12	1.800 (5)	O1_9—C4_9	1.420 (11)
Fe4—C5_13	1.772 (4)	C1_9—C2_9	1.433 (12)
Fe4—C7_13	1.779 (4)	C2_9—C3_9	1.434 (14)
Fe4—C6_13	1.787 (4)	C3_9—C4_9	1.441 (12)
Fe4—C1_13	1.997 (4)	O1_10—C1_10	1.393 (11)
Si1—O2_11	1.651 (2)	O1_10—C4_10	1.433 (11)
Si1—O1_11	1.661 (2)	C1_10—C2_10	1.437 (12)
Si1—C2	1.841 (4)	C2_10—C3_10	1.439 (13)
Si1—C1	1.849 (4)	C3_10—C4_10	1.434 (12)
Fe5—O52	2.073 (2)	O1_12—C1_12	1.146 (4)
Fe5—O51	2.084 (2)	O2_12—C2_12	1.130 (5)
Fe5—O1_2	2.136 (2)	O3_12—C3_12	1.140 (5)
Fe5—O1_3	2.137 (2)	O4_12—C4_12	1.148 (5)
Fe5—O1_4	2.148 (2)	O5_12—C5_12	1.146 (5)
Fe5—O1_1	2.152 (2)	O6_12—C6_12	1.139 (5)
O1_1—C4_1	1.417 (4)	O7_12—C7_12	1.126 (5)
O1_1—C1_1	1.433 (4)	O8_12—C8_12	1.138 (5)
C1_1—C2_1	1.437 (7)	O1_13—C1_13	1.170 (4)
C2_1—C3_1	1.426 (7)	O2_13—C2_13	1.150 (4)
C3_1—C4_1	1.465 (6)	O3_13—C3_13	1.143 (5)
O1_2—C4_2	1.428 (4)	O4_13—C4_13	1.143 (5)
O1_2—C1_2	1.449 (4)	O5_13—C5_13	1.148 (4)
C1_2—C2_2	1.468 (5)	O6_13—C6_13	1.143 (5)
C2_2—C3_2	1.490 (5)	O7_13—C7_13	1.138 (5)
			
O1_11—Ga1—Cl2	106.40 (7)	C5_13—Fe4—Fe2	127.73 (12)
O1_11—Ga1—Cl1	109.33 (7)	C7_13—Fe4—Fe2	117.73 (14)
Cl2—Ga1—Cl1	104.40 (6)	C6_13—Fe4—Fe2	110.59 (13)
O1_11—Ga1—Fe1	98.06 (6)	C1_13—Fe4—Fe2	46.57 (10)
Cl2—Ga1—Fe1	119.97 (4)	Ga2—Fe4—Fe2	56.162 (15)
Cl1—Ga1—Fe1	117.73 (4)	Ga3—Fe4—Fe2	56.193 (17)
O1_11—Ga1—Ga2	43.67 (6)	O2_11—Si1—O1_11	105.74 (11)
Cl2—Ga1—Ga2	127.30 (4)	O2_11—Si1—C2	110.04 (15)
Cl1—Ga1—Ga2	124.73 (4)	O1_11—Si1—C2	109.20 (15)
Fe1—Ga1—Ga2	54.454 (15)	O2_11—Si1—C1	109.79 (16)
O1_11—Ga2—Fe4	115.06 (6)	O1_11—Si1—C1	110.70 (15)
O1_11—Ga2—Fe2	114.81 (6)	C2—Si1—C1	111.2 (2)
Fe4—Ga2—Fe2	67.820 (18)	O52—Fe5—O51	178.40 (10)
O1_11—Ga2—Fe1	91.49 (6)	O52—Fe5—O1_2	86.37 (9)
Fe4—Ga2—Fe1	136.32 (2)	O51—Fe5—O1_2	93.77 (9)
Fe2—Ga2—Fe1	133.09 (2)	O52—Fe5—O1_3	94.26 (10)
O1_11—Ga2—Ga1	40.30 (5)	O51—Fe5—O1_3	87.33 (9)
Fe4—Ga2—Ga1	144.408 (19)	O1_2—Fe5—O1_3	87.01 (10)
Fe2—Ga2—Ga1	138.707 (18)	O52—Fe5—O1_4	91.07 (10)
Fe1—Ga2—Ga1	51.240 (14)	O51—Fe5—O1_4	88.83 (9)
O2_11—Ga3—Fe2	117.53 (6)	O1_2—Fe5—O1_4	177.20 (9)
O2_11—Ga3—Fe4	116.98 (6)	O1_3—Fe5—O1_4	92.06 (10)
Fe2—Ga3—Fe4	67.584 (19)	O52—Fe5—O1_1	89.11 (9)
O2_11—Ga3—Fe3	91.57 (6)	O51—Fe5—O1_1	89.29 (9)
Fe2—Ga3—Fe3	131.33 (2)	O1_2—Fe5—O1_1	92.29 (10)
Fe4—Ga3—Fe3	134.45 (2)	O1_3—Fe5—O1_1	176.50 (9)
O2_11—Ga3—Ga4	40.33 (6)	O1_4—Fe5—O1_1	88.79 (10)
Fe2—Ga3—Ga4	140.748 (19)	C4_1—O1_1—C1_1	107.6 (3)
Fe4—Ga3—Ga4	145.030 (19)	C4_1—O1_1—Fe5	127.0 (2)
Fe3—Ga3—Ga4	51.278 (16)	C1_1—O1_1—Fe5	124.9 (2)
O2_11—Ga4—Cl3	106.92 (7)	O1_1—C1_1—C2_1	106.1 (4)
O2_11—Ga4—Cl4	107.33 (7)	C3_1—C2_1—C1_1	109.5 (5)
Cl3—Ga4—Cl4	103.94 (5)	C2_1—C3_1—C4_1	102.8 (4)
O2_11—Ga4—Fe3	95.80 (6)	O1_1—C4_1—C3_1	108.3 (4)
Cl3—Ga4—Fe3	120.45 (4)	C4_2—O1_2—C1_2	108.1 (3)
Cl4—Ga4—Fe3	120.70 (4)	C4_2—O1_2—Fe5	126.7 (2)
O2_11—Ga4—Ga3	42.18 (6)	C1_2—O1_2—Fe5	125.2 (2)
Cl3—Ga4—Ga3	123.88 (4)	O1_2—C1_2—C2_2	107.1 (3)
Cl4—Ga4—Ga3	127.23 (4)	C1_2—C2_2—C3_2	105.3 (3)
Fe3—Ga4—Ga3	53.649 (17)	C4_2—C3_2—C2_2	102.9 (3)
C5_12—Fe1—C8_12	156.19 (17)	O1_2—C4_2—C3_2	106.1 (3)
C5_12—Fe1—C6_12	101.2 (2)	C1_3—O1_3—C4_3	108.5 (3)
C8_12—Fe1—C6_12	96.6 (2)	C1_3—O1_3—Fe5	127.3 (2)
C5_12—Fe1—C7_12	95.0 (2)	C4_3—O1_3—Fe5	123.6 (2)
C8_12—Fe1—C7_12	96.18 (18)	O1_3—C1_3—C2_3	107.4 (4)
C6_12—Fe1—C7_12	102.99 (19)	C3_3—C2_3—C1_3	109.7 (4)
C5_12—Fe1—Ga1	82.98 (13)	C2_3—C3_3—C4_3	107.4 (4)
C8_12—Fe1—Ga1	83.20 (11)	O1_3—C4_3—C3_3	107.0 (4)
C6_12—Fe1—Ga1	84.40 (13)	C4_4—O1_4—C1_4	106.3 (3)
C7_12—Fe1—Ga1	172.60 (14)	C4_4—O1_4—Fe5	125.6 (3)
C5_12—Fe1—Ga2	78.62 (13)	C1_4—O1_4—Fe5	127.6 (2)
C8_12—Fe1—Ga2	79.02 (12)	O1_4—C1_4—C2_4	104.6 (4)
C6_12—Fe1—Ga2	158.59 (13)	C3_4—C2_4—C1_4	107.4 (5)
C7_12—Fe1—Ga2	98.32 (14)	C2_4—C3_4—C4_4	101.3 (5)
Ga1—Fe1—Ga2	74.306 (17)	O1_4—C4_4—C3_4	109.3 (4)
C2_13—Fe2—C4_13	95.51 (18)	C1_5—O1_5—C4_5	107.9 (3)
C2_13—Fe2—C3_13	100.75 (16)	O1_5—C1_5—C2_5	107.6 (3)
C4_13—Fe2—C3_13	102.41 (19)	C3_5—C2_5—C1_5	106.9 (4)
C2_13—Fe2—C1_13	170.91 (16)	C2_5—C3_5—C4_5	104.4 (4)
C4_13—Fe2—C1_13	87.43 (18)	O1_5—C4_5—C3_5	106.9 (3)
C3_13—Fe2—C1_13	86.97 (17)	C4_6—O1_6—C1_6	107.4 (10)
C2_13—Fe2—Ga2	81.69 (11)	O1_6—C1_6—C2_6	106.9 (11)
C4_13—Fe2—Ga2	170.19 (15)	C1_6—C2_6—C3_6	104.4 (12)
C3_13—Fe2—Ga2	87.37 (12)	C2_6—C3_6—C4_6	99.6 (13)
C1_13—Fe2—Ga2	93.97 (11)	O1_6—C4_6—C3_6	106.3 (10)
C2_13—Fe2—Ga3	84.93 (12)	C1_7—C2_7—C3_7	106.0 (9)
C4_13—Fe2—Ga3	89.98 (15)	C2_7—C3_7—C4_7	98.8 (12)
C3_13—Fe2—Ga3	165.70 (12)	C4_8—O1_8—C1_8	108.2 (3)
C1_13—Fe2—Ga3	86.47 (12)	O1_8—C1_8—C2_8	107.3 (4)
Ga2—Fe2—Ga3	80.432 (17)	C3_8—C2_8—C1_8	105.6 (4)
C2_13—Fe2—Fe4	124.29 (11)	C2_8—C3_8—C4_8	108.1 (4)
C4_13—Fe2—Fe4	119.81 (14)	O1_8—C4_8—C3_8	107.2 (4)
C3_13—Fe2—Fe4	110.52 (12)	C1_9—O1_9—C4_9	110.5 (8)
C1_13—Fe2—Fe4	47.47 (10)	O1_9—C1_9—C2_9	104.1 (10)
Ga2—Fe2—Fe4	56.018 (16)	C1_9—C2_9—C3_9	107.6 (10)
Ga3—Fe2—Fe4	56.223 (17)	C2_9—C3_9—C4_9	106.1 (10)
C4_12—Fe3—C1_12	154.46 (16)	O1_9—C4_9—C3_9	105.8 (9)
C4_12—Fe3—C3_12	97.93 (19)	C1_10—O1_10—C4_10	108.3 (9)
C1_12—Fe3—C3_12	96.20 (19)	O1_10—C1_10—C2_10	107.3 (9)
C4_12—Fe3—C2_12	98.2 (2)	C1_10—C2_10—C3_10	107.2 (11)
C1_12—Fe3—C2_12	99.2 (2)	C4_10—C3_10—C2_10	104.3 (12)
C3_12—Fe3—C2_12	102.9 (2)	O1_10—C4_10—C3_10	107.3 (11)
C4_12—Fe3—Ga4	80.49 (13)	Si1—O1_11—Ga1	129.22 (11)
C1_12—Fe3—Ga4	80.83 (12)	Si1—O1_11—Ga2	134.72 (11)
C3_12—Fe3—Ga4	166.53 (14)	Ga1—O1_11—Ga2	96.03 (8)
C2_12—Fe3—Ga4	90.51 (14)	Si1—O2_11—Ga4	129.74 (12)
C4_12—Fe3—Ga3	79.55 (13)	Si1—O2_11—Ga3	132.76 (12)
C1_12—Fe3—Ga3	78.92 (12)	Ga4—O2_11—Ga3	97.49 (8)
C3_12—Fe3—Ga3	91.47 (14)	O1_12—C1_12—Fe3	178.8 (3)
C2_12—Fe3—Ga3	165.58 (14)	O2_12—C2_12—Fe3	177.6 (4)
Ga4—Fe3—Ga3	75.072 (17)	O3_12—C3_12—Fe3	178.6 (5)
C5_13—Fe4—C7_13	95.13 (19)	O4_12—C4_12—Fe3	178.9 (3)
C5_13—Fe4—C6_13	97.47 (19)	O5_12—C5_12—Fe1	178.4 (4)
C7_13—Fe4—C6_13	104.4 (2)	O6_12—C6_12—Fe1	176.3 (4)
C5_13—Fe4—C1_13	173.98 (16)	O7_12—C7_12—Fe1	179.3 (5)
C7_13—Fe4—C1_13	87.27 (18)	O8_12—C8_12—Fe1	177.5 (3)
C6_13—Fe4—C1_13	87.25 (17)	O1_13—C1_13—Fe2	138.1 (3)
C5_13—Fe4—Ga2	83.14 (13)	O1_13—C1_13—Fe4	135.9 (3)
C7_13—Fe4—Ga2	167.91 (15)	Fe2—C1_13—Fe4	85.96 (13)
C6_13—Fe4—Ga2	87.65 (14)	O2_13—C2_13—Fe2	175.5 (3)
C1_13—Fe4—Ga2	93.36 (10)	O3_13—C3_13—Fe2	179.0 (4)
C5_13—Fe4—Ga3	88.77 (13)	O4_13—C4_13—Fe2	177.5 (4)
C7_13—Fe4—Ga3	87.52 (15)	O5_13—C5_13—Fe4	173.2 (3)
C6_13—Fe4—Ga3	165.87 (14)	O6_13—C6_13—Fe4	179.0 (4)
C1_13—Fe4—Ga3	85.82 (12)	O7_13—C7_13—Fe4	176.9 (5)
Ga2—Fe4—Ga3	80.497 (17)		

### trans-Diaquatetrakis(tetrahydrofuran-κO)iron(II) µ-carbonyl-tetradecacarbonyltetrachlorido-µ-dimethylsilanediolato-tetragalliumtetrairon(7 Ga–Fe)(Fe–Fe) tetrahydrofuran tetrasolvate Hydrogen-bond geometry (Å, º)

**Table:**

*D*—H···*A*	*D*—H	H···*A*	*D*···*A*	*D*—H···*A*
O51—H51*A*···O1_5	0.96 (2)	1.72 (2)	2.677 (3)	175 (2)
O51—H51*B*···O1_7	0.96 (2)	1.71 (2)	2.623 (11)	159 (2)
O51—H51*B*···O1_6	0.96 (2)	1.71 (2)	2.671 (15)	179 (3)
O52—H52*A*···O1_8	0.96 (2)	1.71 (2)	2.653 (4)	170 (3)
O52—H52*B*···O1_10	0.96 (2)	1.71 (2)	2.665 (12)	175 (3)
O52—H52*B*···O1_9	0.96 (2)	1.71 (3)	2.647 (16)	167 (3)
